# Omixer: multivariate and reproducible sample randomization to proactively counter batch effects in omics studies

**DOI:** 10.1093/bioinformatics/btab159

**Published:** 2021-03-08

**Authors:** Lucy Sinke, Davy Cats, Bastiaan T Heijmans

**Affiliations:** Molecular Epidemiology, Department of Biomedical Data Science, Leiden University Medical Centre, Leiden 2333 ZC, The Netherlands; Molecular Epidemiology, Department of Biomedical Data Science, Leiden University Medical Centre, Leiden 2333 ZC, The Netherlands; Molecular Epidemiology, Department of Biomedical Data Science, Leiden University Medical Centre, Leiden 2333 ZC, The Netherlands

## Abstract

**Motivation:**

Batch effects heavily impact results in omics studies, causing bias and false positive results, but software to control them preemptively is lacking. Sample randomization prior to measurement is vital for minimizing these effects, but current approaches are often ad hoc, poorly documented and ill-equipped to handle multiple batches and outcomes.

**Results:**

We developed Omixer—a Bioconductor package implementing multivariate and reproducible sample randomization for omics studies. It proactively counters correlations between technical factors and biological variables of interest by optimizing sample distribution across batches.

**Availabilityand implementation:**

Omixer is available from Bioconductor at http://bioconductor.org/packages/release/bioc/html/Omixer.html. Scripts and data used to generate figures available upon request.

**Supplementary information:**

Supplementary data are available at *Bioinformatics* online.

## 1 Introduction

Batch effects can overshadow biological differences in size (Baggerly *et al.*, 2008) and critically influence the results of omics studies (Harper *et al.*, 2013; Lambert and Black, 2012). Even in benign cases, they decrease power to detect a true biological effect or contaminate results with false positives (Leek *et al.*, 2010). Despite the numerous statistical methods developed to adjust for batch effects (Espin-Perez *et al.*, 2018; Johnson *et al.*, 2007; van Iterson *et al.*, 2017), a reactive approach is often insufficient. In fact, when technical variables are confounded with experimental factors of interest, batch effect correction will mask the underlying biological signal (Goh *et al.*, 2017).

Sample randomization is a proactive, and arguably more impactful, method for obtaining reproducible results in high-throughput experiments (Yang *et al.*, 2008). However, its implementation suffers from several key issues. Particularly where there are numerous or nested batches each composed of a limited number of samples, such as separate microarrays or sequencing lanes, single random draws can inadvertently result in high correlations between technical covariates and biological factors. This is further complicated by an often poorly documented randomization process that is not necessarily reproducible. Although stratified randomization has been shown to effectively remove chip effects in microarray experiments (Buhule *et al.*, 2014), it does not address all relevant biological variables. Therefore, to adequately combat bias in results, employing methods capable of handling a wider array of research setups is imperative.

We developed Omixer—an R package for multivariate and reproducible randomization in omics studies. From a diverse range of randomized sample layouts, it selects the one that optimally balances biological variables across batches. Omixer offers the flexibility required to perform randomization effectively in a variety of study designs and experimental setups.

## 2 Materials and methods

**Fig. 1.**
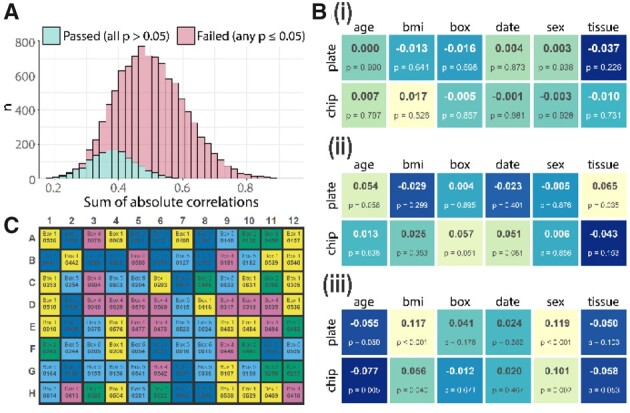
Overview of Omixer functionality and graphical output with (**A**) distribution of the sum of absolute correlations from 10 000 randomized layouts, coloured by filtering step outcome (**B**) resulting correlation matrices from the (i) optimal Omixer layout, (ii) median result and (iii) worst case scenario after simple randomization and (**C**) lab-friendly sample sheets created by Omixer as a PDF, showing the first plate colour coded by box number

To optimize distribution of biological variables across batches, sample randomization is performed multiple times (default: 1000; see Supplementary Fig. S1 for more information). After combining resulting lists with the user-specified plate layout, statistically robust tests of correlation determine the optimal setup, where the absolute sum of correlations between biological and technical factors is minimized. As a precautionary step, layouts with evidence for any tested batch associations are excluded (*P* < 0.05 for any batch-outcome correlation), although in practice this will not change the resulting layout given suitably large iteration numbers (see Fig. 1A).

To reserve wells for control samples or other studies, a mask can be specified in the options, and paired samples such as those from twin studies can be blocked so they remain together in the same batch. Non-standard plate layouts can be specified, but Omixer will automatically generate the most commonly used plate and chip combinations. Previously generated layouts can quickly be reproduced, and lab-friendly sample sheets reduce the risk of mixups when manually pipetting samples.

### 2.1 Multivariate and reproducible randomization

The main function, omixerRand, takes a sample list and plate layout as input and optimizes distribution of specified biological variables across batches. Resulting correlations are visually displayed and the optimal seed is saved locally. By loading this seed, previously generated layouts can be reproduced quickly and efficiently with the omixerSpecific function.

### 2.2 Lab-friendly sample sheets

The omixerSheet function converts the output of previous Omixer functions into lab-friendly sample sheets, saving these in the working directory as a printable PDF. Wells can be coloured by other variables, such as box number (see Fig. 1C) or tissue, to further smooth transition into the wet lab.

### 2.3 Omixer outperforms simple randomization

Particularly when multiple batch types and outcomes are present, a single randomization is likely to result in significant correlations. As an example, we randomized 672 samples across 2 levels of batches, as described in the Omixer vignette. Following 10 000 simple randomizations, 85% of the resulting layouts have at least one *P*-value under 0.05. The distribution of the sum of absolute correlations for the resulting 10 000 layouts (Fig. 1A) suggests that the expected sum of correlations between batches and outcomes following a single randomization is 0.5. The correlations present in an average selection (Fig. 1B.ii) are small on the whole (0.004 to 0.065), but significant associations (*P* < 0.05) still exist.

Looking at the worst case scenario following simple randomization (Fig. 1B.iii), we see that simple randomization has the potential to choose layouts with multiple significant associations (*P* < 0.05 for 5 comparisons), resulting in large batch effects that will bias results. By contrast, Omixer would reject all layouts with significant correlations, and instead return an optimal layout from the 15% remaining (blue in Fig. 1A). In this example, the optimal layout’s correlations (Fig. 1B.i) are all under 0.037 and none are significant.

## 3 Conclusions

In conclusion, Omixer offers an intuitive, reproducible alternative to current randomization practices in omics research. Its implementation is a key step in combatting batch effects preemptively and reducing the risks of sample mixups in the wet lab.

## Funding

This work was supported by the Joint Programming Initiative ‘a Healthy Diet for a Healthy Life’ (JPI-HDHL) DIMENSION project [ZonMW project number: 529051021].

*Conflict of Interest*: none declared.

## Data availability

Scripts and data used to generate the images is available upon request. Otherwise, Omixer is a software tool that uses user input data.

## Supplementary Material

btab159_Supplementary_Data
