# Supplementary material for: Omixer: multivariate and reproducible sample randomization to proactively counter batch effects in omics studies
Source: Bioinformatics. 2021 Mar 8;37(18):3051–2. doi: 10.1093/bioinformatics/btab159 (PMC10262301; doi:10.1093/bioinformatics/btab159)

Omixer: multivariate and reproducible sample randomization to proactively counter batch effects in omics studies

Supplementary Information

The default number of iterations Omixer performs is 1,000. Increasing this number may result in smaller overall correlations, and complex designs could benefit from increases above the default. However, users should be aware that this choice comes at the cost of increased runtime. This figure shows the runtime using 1CPU with 64GB RAM to randomize the extended example samples described in the Omixer vignette. This may prove useful to users in determining an appropriate number of iterations for their situation.

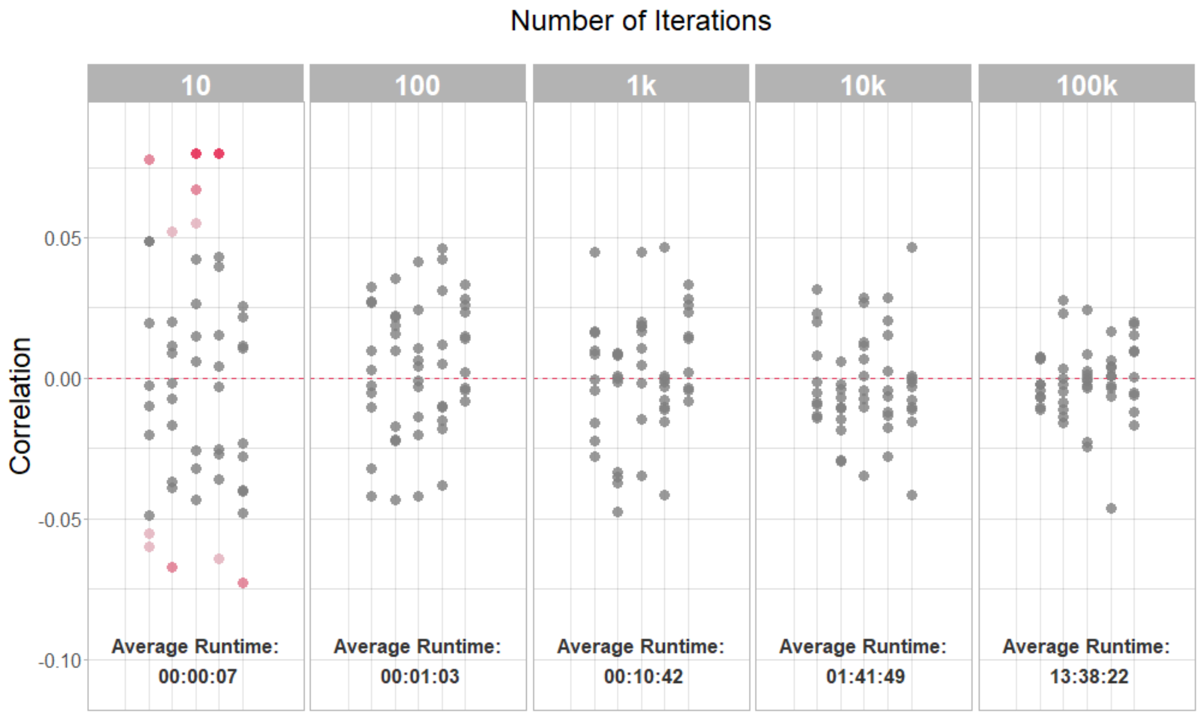

Supplement: btab159_Supplementary_Data [file btab159_supplementary_data.zip › Sinke-Omixer_Bioinformatics-Supplementary-Final-Template.pdf]
